# Supplementary material for: A synthetic biosensor to detect peroxisomal acetyl-CoA concentration for compartmentalized metabolic engineering
Source: PeerJ. 2020 Sep 8;8:e9805. doi: 10.7717/peerj.9805 (PMC7485502; doi:10.7717/peerj.9805)
Supplement: Supplemental Information 1 [file peerj-08-9805-s001.docx]

Supporting Information

**For: A Synthetic Biosensor to Detect Peroxisomal Acetyl-CoA Concentration for Compartmentalized Metabolic Engineering**

Herbert M. Huttanus^1^

Ryan S. Senger^1,2^

^1^Department of Biological Systems Engineering, Virginia Polytechnic Institute and State University, USA

^2^Department of Chemical Engineering, Virginia Polytechnic Institute and State University, USA

Table S1: Plasmids and primers.

| **Plasmids used in this study** | |  |
| --- | --- | --- |
| Name | Description |  |
| pKK01 | Ura3-(adh1t-phaA-pgk1p)-(tef1p-phaB-cyc1t)-Amp-(tef1p-phaC-cyc1t) | |
| pRS416 | Ura3-Amp |  |
| pABc | pRS416-(adh1t-phaA-pgk1p)-(tef1p-phaB-cyc1t) | |
| pABp | pRS416-(adh1t-ePTS1-phaA-pgk1p)-(tef1p-phaB-ePTS1-cyc1t) | |
| pCc | pRS416-(pdc1p-phaC-pdc1t) |  |
| pCp | pRS416-(pdc1p-phaC-ePTS1 pdc1t) |  |
| pPHBc | pRS416-(adh1t-phaA-pgk1p)-(tef1p-phaB-cyc1t)-(pdc1p-phaC-pdc1t) | |
| pPHBp | pRS416-(adh1t-ePTS1-phaA-pgk1p)-(tef1p-phaB-ePTS1-cyc1t)-(pdc1p-phaC-ePTS1 pdc1t) | |
| **Strains used in this study** | |  |
| Name | Phenotype | Plasmids |
| BY4741 | MATa his3Δ1 leu2Δ0 met15Δ0 ura3Δ0 |  |
| INVSc1 | MATa his3Δ1 leu2Δ0 trp1-289 ura3-52 |  |
| phbC | Same as BY4741 | pPHBc |
| phbP | Same as BY4741 | pPHBp |
| PΔaat2 | BY4741Δaat2 | pPHBp |
| PΔadr2 | BY4741Δadr2 | pPHBp |
| PΔrpd3 | BY4741Δrpd3 | pPHBp |
| PΔsin3 | BY4741Δsin3 | pPHBp |
| PΔpot1 | BY4741Δpot1 | pPHBp |
| PΔpox1 | BY4741Δpox1 | pPHBp |
| PΔpex5 | BY4741Δpex5 | pPHBp |
| PΔpex7 | BY4741Δpex7 | pPHBp |
| PΔopi1 | BY4741Δopi1 | pPHBp |
| PΔinp2 | BY4741Δinp2 | pPHBp |
| PΔslt2 | BY4741Δslt2 | pPHBp |
| PΔatg36 | BY4741Δatg36 | pPHBp |
| PΔino1 | BY4741Δino1 | pPHBp |
| **Primers used in this study** | |  |
| Name | Sequence |  |
| 1: pRS_adh1t_f | cgacggtatcgataagcttgatatcgaattcctgcagcccgagcgacctcatgctatacc | |
| 2: pRS_cyc1t_r | ccaccgcggtggcggccgctctagaactagtggatccccccttcgagcgtcccaaaacct | |
| 3: phaA_adh1t_rp | ggaaagaaagctggggcgtggccgtaggtcaaaattatgagagctcttaattaacaattc | |
| 4: adh1t_phA_fp | ttaagagctctcataattttgacctacggccacgccccagctttctttccaccgccaatg | |
| 5: cyc1t_phaB_rp | ccatgtcgacctataattttgacctacggccacgccccagacccatgtgtagaccaccgt | |
| 6: phaB_cyc1t_fp | acacatgggtctggggcgtggccgtaggtcaaaattataggtcgacatggaacagaagtt | |
| 7: phaC_pdc1p_r | agatgcagcagcacctttaccagttgccattttgtttgaatttgattgatttgactgtgt | |
| 8: pRS_pdc1p_f | cgacggtatcgataagcttgatatcgaattcctgcagccccatgcgactgggtgagcata | |
| 9: pdc1p_phaC_f | tcataacctcacgcaaaataacacagtcaaatcaatcaaattcaaacaaaatggcaactg | |
| 10: pdc1t_phaC_r | tgcttataaaactttaactaataattagagattaaatcgcctatgcttttgccttaacat | |
| 11: phaC_pdc1t_f | agagccggcacccggtaggtatgttaaggcaaaagcataggcgatttaatctctaattat | |
| 12: pRS_pdc1t_r | ccaccgcggtggcggccgctctagaactagtggatcccccggcagttttgaattgagtaa | |
| 13: pdc1t_phaC_rp | attaaatcgcctataattttgacctacggccacgccccagtgcttttgccttaacatacc | |
| 14: phaC_pdc1t_fp | ggcaaaagcactggggcgtggccgtaggtcaaaattataggcgatttaatctctaattat | |
| 15: pdc1p_cyc1t_r | cacatcacatcagcggaacatatgctcacccagtcgcatgcttcgagcgtcccaaaacct | |
| 16: cyc1t_pdc1p_f | actgaaaaccttgcttgagaaggttttgggacgctcgaagcatgcgactgggtgagcata | |

“ePTS1” indicates the addition of the enhanced PTS1 localization tag on the C terminus of the genes. The amino acid sequence of the enhanced tag is LGRGRRSKL. Underlined gene cassettes are in the opposite direction of most of the other genes and are described in the appropriate reverse order.

Extended Methods

All plasmids were constructed by Assembler homologous recombination. Plasmid pABc was constructed using a XmaI-linearized pRS416 backbone and one fragment containing both phaA and phaB genes using primers 1 and 2 with pKK01 as a template. Plasmid pABp required more fragments to introduce the ePTS1 tags and included the pRS416 backbone, adh1t (using primers 1 and 3 with pKK01 as a template), phaA and phaB together (amplified using primers 4 and 5 with pKK01 as a template) and cyc1t (amplified using primers 2 and 6 with pKK01 as a template). Plasmid pCc was constructed from the pRS416 backbone and three PCR generated overlapping fragments. The fragments include pdc1p (using primers and 7 and 8 with an INVSc genomic template), phaC (using primers 9 and 10 with pKK01 as a template) and pcd1t (using primers 11 and 12 with INVSc genomic template). pCp was constructed with the same method except that the ePTS1 tags were introduced by using primers 13 and 14 instead of 10 and 11 respectively. All four of these plasmids were transferred from *S. cerevisiae* to *E. coli* for storage and to generate enough plasmid for proper assembly verification and subsequent steps.

Plasmid pPHBc was constructed using the pRS416 backbone and two fragments. The fragment containing phaA and phaB was amplified from pABc using primers 1 and 15 and the fragment containing phaC was amplified from pCc using primers 12 and 16. Plasmid pPHBp was prepared using the same backbone and primers but using pABp and pCp as templates respectively.
